# Melatonin mitigates PGF-induced apoptosis during luteal regression in heat-exposed rats

**DOI:** 10.1590/1984-3143-AR2024-0122

**Published:** 2025-06-30

**Authors:** Hadi Tavakolikazerooni, Hao Yu, Saif Ullah, Wael Ennab, Dagan Mao

**Affiliations:** 1 College of Animal Science and Technology, Nanjing Agricultural University, Nanjing, China; 2 College of Animal Science and Technology, Yangzhou University, Yangzhou, PR China

**Keywords:** heat stress, luteal regression, apoptosis, melatonin

## Abstract

This study investigates the protective effects of melatonin against heat exposure during PGF-induced luteal regression in rats. Seventy-five PMSG and hCG primed rats were divided into three groups: non-heat-exposure (NHE), heat-exposure (HE), and melatonin *plus* heat-exposure (M+HE). The HE group underwent daily heat exposure (41°C for 2 h) for 7 days, while the M+HE group received intraperitoneal injection of melatonin (10 mg/kg body weight) before each heat session. On Day 7, PGF was administered, and ovarian samples were collected at 0, 1, 2, 8, and 24 h post-PGF. One set of ovaries was processed for histological analysis, including H&E staining, immunohistochemistry, and transmission electron microscopy and the other set was processed for Western blot for apoptotic protein expression. Results showed that heat exposure increased ovarian weight, disrupted follicular development, and elevated ovarian apoptotic markers (Caspase-3 and Bax), leading to luteal cell damage. Melatonin preserved ovarian weight, improved follicular and luteal structure, reduced atretic follicles, and mitigated luteal cell degeneration. In addition, melatonin decreased apoptotic marker expression and the Bax/Bcl-2 ratio, particularly at 16 and 24 h. These findings suggest melatonin protects luteal cells from heat-induced apoptosis during PGF-triggered regression, supporting reproductive function.

## Introduction

The corpus luteum (CL) is a key regulator of female reproductive health, primarily responsible for maintaining early pregnancy through the secretion of progesterone (P4), which is essential for sustaining pregnancy and promoting embryo development (Boni, 2019; Collier et al., 2017; Ullah et al., 2019).

Heat stress is a significant environmental factor that disrupts reproductive function by impairing CL function, progesterone synthesis and contributing to infertility (Abate et al., 2020; Agarwal et al., 2012; Nanas et al., 2021; Wolfenson et al., 2002). It accelerates and dysregulates luteal regression, leading to both functional impairment and structural damage in luteal cells through enhanced apoptosis (Hojo et al., 2022).

Apoptosis, a tightly regulated form of programmed cell death, plays a crucial role in CL regression under heat stress conditions (Hojo et al., 2022; Lockshin and Zakeri, 2001). Heat stress disrupts mitochondrial function, leading to the release of cytochrome c, which activates the intrinsic apoptotic pathway. This pathway is initiated by the upregulation of pro-apoptotic proteins, which promote mitochondrial outer membrane permeabilization, leading to caspase activation and subsequent cell death (Asadi et al., 2022). Caspase-3, a key effector, mediates apoptosis by cleaving key cellular substrates, involved in DNA repair and cell survival. This pathway is regulated by Bcl-2 family proteins, which control mitochondrial permeability and determine the fate of luteal cells (Khan et al., 2020a; Sugino et al., 2000; Warren et al., 2019). In addition, the pro-apoptotic members such as Bax and Bak tipping the balance toward cell death, while anti-apoptotic proteins like Bcl-2 and Bcl-xL counteract this effect (Kumariya et al., 2021). The interplay between these proteins determines luteal cell fate and plays a critical role in heat stress-induced luteolysis.

Melatonin, a neurohormone primarily produced by the pineal gland, exhibits strong antioxidant and anti-apoptotic properties (Sun et al., 2020). It modulates key regulators of apoptosis, including Bax, Bcl-2 (Mohseni et al., 2012), and Caspases (Sainz et al., 2003), affecting both intrinsic and extrinsic apoptotic pathways. These mechanisms make melatonin a promising candidate for mitigating heat stress-induced apoptosis, particularly in reproductive tissues (Hassan et al., 2022). Its ability to neutralize reactive oxygen species and reduce cellular damage and apoptosis (Andrabi et al., 2004; Manchester et al., 2015). In luteal cells, these protective mechanisms suggests that melatonin may mitigate heat stress-induced apoptosis, with potential implications for reproductive health.

This study hypothesized that melatonin modulate luteal cell apoptosis in rats subjected to heat stress. By using PMSG/hCG treatment to induce mature corpora lutea in immature rats, we investigated luteal structure and apoptosis through the analysis of apoptotic proteins and subcellular structures. The findings provide valuable insights into the interaction between melatonin, heat stress and ovarian function, offering potential strategies to improve infertility treatments.

## Methods

All experimental procedures were approved by the Institutional Animal Care and Use Committee of Nanjing Agricultural University, China, with approval numbers: SYXK2022-0031.

### Animals

Three-week-old female Sprague-Dawley rats (50 ± 5 g), specific pathogen-free (SPF), were obtained from the Laboratory Animal Center of Nanjing Agricultural University, Nanjing, China. Rats were housed in standard cages at 25°C, under a 12-h light-dark cycle, with ad libitum access to food and water.

### Experimental design and treatment

After a 7-day acclimation period, 75 rats aged 28 days were injected intraperitoneally with 30 IU of pregnant mare’s serum gonadotropin (PMSG, Ningbo Sansheng Pharmaceutical, Zhejiang, China) was administered to stimulate follicular development, which corresponds to the proestrus phase, followed by an injection of 30 IU human chorionic gonadotropin 48 h later (hCG, Ningbo Sansheng Pharmaceutical) to induce ovulation and establish a single generation of CL, which corresponds to the estrus phase, as previously described (Bai et al., 2017; Chen et al., 1999; Guo et al., 2015; Pan et al., 2004; Qi et al., 2018; Tang et al., 2021).

The day of hCG injection was designated as Day 0. 24 h post-hCG, the rats enter the metestrus phase, which marks the beginning of the luteal phase. The rats were then divided into three groups (n = 25 per group). *Non-heat exposure* (NHE): housed under standard conditions. *Heat exposure* (HE): exposed to heat (41°C, 2 h daily, 11 AM to 1 PM) for 7 consecutive days. Heat stress was induced using temperature-controlled chambers (Bai et al., 2017; Liu et al., 2014; Yu et al., 2011). *Melatonin plus heat exposure* (M+HE): exposed to the same heat regimen as the HE group, with melatonin (Cat. No.: HY-B0075, MedChemExpress) dissolved in 0.9% saline with 1% ethanol and administered intraperitoneally 1 hour prior to heat exposure, daily for 7 days at a dose of 10 mg/kg body weight was selected based on previous studies (Mojaverrostami et al., 2019; Reiter et al., 2016).

On Day 7, following a 2-h post-stress recovery period, rats were administered either 0.3 mL of PGF (Cloprostenol, a synthetic PGF_2α_ analogue, 0.1 mg/mL, Ningbo Sansheng Pharmaceutical) or 0.9% saline (0 time point) intraperitoneally, to induces luteal regression, mimicking the natural regression process. The rats were euthanized at 0, 2, 8, 16, or 24 h post-PGF injection under ether anesthesia. At each time point, ovaries were collected. One ovary was fixed in 4% paraformaldehyde and then embedded in paraffin wax for hematoxylin and eosin (H&E) staining, immunohistochemistry (IHC), or in 2.5% glutaraldehyde for transmission electron microscopy (TEM) analysis, whereas the other was stored at -80°C for western blot (WB) analysis.

### Physiological monitoring

Food intake, and water consumption were monitored daily in each group (n = 25) and recorded prior to heat exposure session. Rectal temperature (Tc) was recorded daily to confirm stress induction, using a thermocouple (BW-TH1101, Shanghai, China) by inserting a thermometer 1.5 cm into the rectum, and the maximum temperature (Tc, max) was documented both before and after heat exposure for each group (n = 25) during the 7-day period.

### Body weight and ovarian weight measurement

Body weight was measured daily (n = 25 per group) and recorded immediately before euthanasia at the end of the experimental period. Ovaries were carefully dissected and weighed separately from the body. The ovarian index was calculated as the ratio of ovarian weight to body weight, expressed as a percentage.

### Histological analysis

Ovarian samples were fixed in 4% paraformaldehyde, dehydrated in graded ethanol, cleared in xylene, and embedded in paraffin wax. Sections (5 μm thick) were stained with H&E for histological evaluation, as previously described (Li et al., 2024). Light microscopy (Olympus Optical Co., Ltd., Beijing, China) was employed to observe morphological changes in the corpus luteum and identify potential signs of apoptosis, including nuclear condensation, chromatin fragmentation, and the presence of apoptotic bodies (Alchalabi et al., 2016). These observations are qualitative and descriptive, without statistical analysis.

### Follicle counting

Ovaries collected at 0 h post-PGF injection were used to assess follicular development. After fixation in 4% formaldehyde, the ovaries underwent standard histological processing, including dehydration in ethanol, clearing in xylene, and embedding in paraffin. Serial sections (5 μm thick) were taken from every fifth section and stained with H&E for morphological assessment. Normal follicles were classified into four categories: Primordial Follicles: Single layer of tabular cells; Primary Follicles: One to two layers of cuboidal cells; Secondary Follicles: Two to six layers of cuboidal cells; Tertiary Follicles: More than six layers of squamous cells, with presence of cumulus oophorus, antrum formation. Atretic follicles: Identified by disorganized granulosa cell layers, a pyknotic oocyte nucleus, eosinophilic cellular debris, irregular follicular boundaries. Follicles were counted only if a visible oocyte nucleus was present, and the counts were multiplied by five to estimate total follicle numbers. Counts were performed on every fifth section to avoid double-counting (Chakravarthi et al., 2021).

### Western blot analysis

Western blot was performed as previously described (Yu et al., 2023). Ovaries were homogenized in radio-immuno-precipitation assay (RIPA) buffer (Beyotime, Nantong, China) containing 10 mM phenylmethylsulphonyl fluoride (PMSF; Beyotime). Total proteins were obtained by centrifugation of samples at 12,000 g for 20 minutes at 4°C. The resulting pellets were discarded. The bicinchoninic acid (BCA) assay kit (Beyotime Institute of Biotechnology, Jiangsu, China) was used to measure protein content, and equal amounts of protein were loaded onto 10% sodium dodecyl sulfate (SDS)–polyacrylamide gels. Proteins were then transferred to polyvinylidene difluoride (PVDF) membranes (Millipore, Bedford, MA, USA) and blocked with 5% bovine serum albumin (BSA) in Tris-buffered saline Tween-20 (TBST) for 90 minutes at room temperature (15-25°C). Subsequently, membranes were incubated with specific primary antibodies (Table 1) overnight at 4°C. Following three washes for 5 minutes each with TBST, membranes were incubated with appropriate horseradish peroxidase-conjugated secondary antibodies (Table 1) for 2 h at room temperature. Finally, membranes were detected using an enhanced chemiluminescence (ECL) system (Chemistar Highsig ECL Western Blotting Substrate; Tanon, Nanjing, China). Protein bands were visualized using a Luminescent Image Analyzer LAS4000 (Fuji Film, Tokyo, Japan), and the integrated light intensity for each band was quantified using ImageJ software (ImageJ, version 1.54d, National Institutes of Health, Bethesda, MD, USA).

**Table 1:** Antibodies used for western blot and immunohistochemistry.

**Antibodies**	**CAT No.**	**Supplier**	**Antigen Source**	**Host**	**Dilution WB**	**Dilution IHC**
α-Tubulin	AC007	Abclonal	Human	Rabbit	1: 3000	---
β-Actin	AC004	Abclonal	Human	Mouse	1: 5000	---
Bax	A0207	Abclonal	Human	Rabbit	1: 1000	1:100
Bcl-2	A0208	Abclonal	Human	Rabbit	1: 1000	---
Caspase-3	A16793	Abclonal	Human	Rabbit	1: 1000	1:100
HRP Anti-M IgG	AS014	Abclonal	Rabbit	Goat	1: 10000	---
HRP Anti-R IgG	AS003	Abclonal	Mouse	Goat	1: 10000	---

### Transmission electron microscopy

Transmission electron microscopy was performed as previously described (Ullah et al., 2019). The ovarian luteal tissues were initially fixed using 2.5% glutaraldehyde in PBS (pH 7.4) at 4 °C for 3 h at room temperature. Subsequently, the tissues were washed twice with PBS for 15 minutes each, followed by fixation with 1% osmium tetroxide (75632 Sigma, Sigma-Aldrich Merck, UK) in PBS for 2 h. Afterward, the samples were washed with deionized water. After dehydration in ethyl alcohol through ascending concentration, the dehydrated tissues were embedded in Araldite. Tissue blocks were then sliced into thin sections (0.06 μm) using a Reichert ultramicrotome (ultra cut E) and mounted on copper grids. These sections were stained with Reynolds lead citrate and uranyl acetate. The stained sections were observed, photographed, and examined using a Philips CM-120 electron microscope (Philips/FEI, The Netherlands). The ultrastructural features of the CL, including apoptotic bodies, autophagosomes, mitochondrial morphology and lipid droplet accumulation, were examined.

### Immunohistochemistry

Immunohistochemistry was performed as previously described (Yu et al., 2023). Briefly, ovary samples embedded in paraffin wax were sectioned into 5-μm slices. Sections were then deparaffinized in xylene, followed by rehydration through a descending series of ethanol solutions. Subsequently, endogenous peroxidase activity was nullified by treatment with 0.3% H_2_O_2_. To mitigate non-specific binding, sections were blocked with 5% BSA for 1 h at room temperature. Sections were subjected to overnight incubation at 4°C with primary antibodies (Table 1). Specific protein immunoreactivity was visualized using a streptavidin biotin - peroxidase complex (SABC) kit (Boster Biological Technology, Wuhan, China). Sections were then stained with 3,3-diaminobenzidine (DAB; Sigma-Aldrich, St Louis, MO, USA) to serve as the chromogen. As a negative control, PBS was employed instead of the primary antibody. The images were captured using a Nikon YS100 microscope (Nikon, Tokyo, Japan) equipped with a digital camera (Model D3300; Nikon) and ImageJ software (ImageJ, version 1.54d, National Institutes of Health, Bethesda, MD, USA), which featured an IHC profiler compatible plug-in, was used for analysis.

### Statistical analysis

All data are presented as means ± standard error of the mean (SEM). Statistical analyses were performed using SPSS software, version 17.0 (SPSS Inc., Chicago, IL, USA). Comparisons within groups across different time points or among groups at the same time points post-PGF treatment were evaluated using one-way analysis of variance (ANOVA), followed by Tukey’s post-hoc test for multiple comparisons. In case of non-normality, data distribution was assessed using the Shapiro-Wilk test. For non-normally distributed data, the Kruskal-Wallis test was used for non-parametric comparisons. A *p*-value of less than 0.05 (*p* < 0.05) was considered statistically significant.

## Results

### Effects of melatonin on physiological responses of rats during heat stress

**Figure 1:**
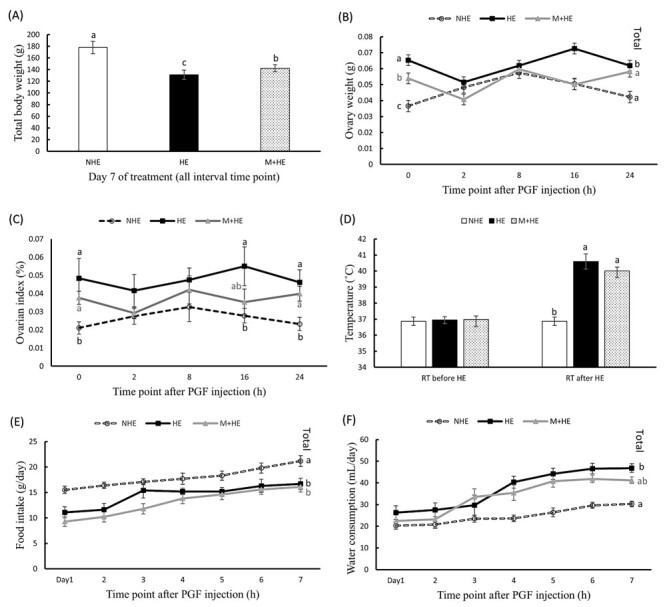
Effects of melatonin on growth and physiological responses in heat-exposed rats. Rats were treated with or without heat exposure (41°C, 2 h) and melatonin (10 mg/kg body weight) for 7 days. Measurements include rectal temperature recorded before and after heat exposure, daily feed intake, and water consumption. On Day 7, rats received PGF_2α_ (0.1 mg/mL) or saline and were euthanized for ovarian analysis. Panels show (A) total body weight, (B) ovary weight, (C) ovarian index, (D) rectal temperature, (E) daily food intake, and (F) daily water consumption. Data are presented as mean ± SEM (n = 25 for each group). Different letters indicate significant differences (*p* < 0.05).

Heat exposure significantly impaired animal growth, as evidenced by reduction in total body weight in the HE group after 7 days of exposure (*p* < 0.05). However, melatonin treatment partially mitigated this effect, resulting in a significantly higher body weight in the M+HE group compared with the HE group (*p* < 0.05, Figure 1A). Ovarian weight in the HE group was significantly higher at 0 h and total ovarian weight compared with NHE group (*p* < 0.05, Figure 1B). However, melatonin treatment alleviated this increase, significantly reducing ovarian weight at both 0 h and overall (*p* < 0.05, Figure 1B).

The ovarian index remained unchanged within individual groups over time. However, between groups, the heat exposure exhibited a significant increase in the ovarian index in HE group at 0, 16, and 24 h and in M+HE group at 0 and 24 h compared with the NHE group (*p* < 0.05, Figure 1C).

Rectal temperatures were monitored throughout the 7-day heat exposure period. A marked increase in rectal temperature was observed after each day of heat treatment (*p* < 0.05, Figure 1D).

Food intake significantly decreased in both HE and M+HE groups compared with the NHE group (*p* < 0.05, Figure 1E). Meanwhile, water consumption significantly increased in the HE group after 7 days of heat exposure (*p* < 0.05, Figure 1F). However, no significant differences in water consumption or food intake were detected between the HE and M+HE groups (Figure 1E and F).

### Effects of melatonin on ovarian follicles and morphology of rats under heat stress

**Figure 2:**
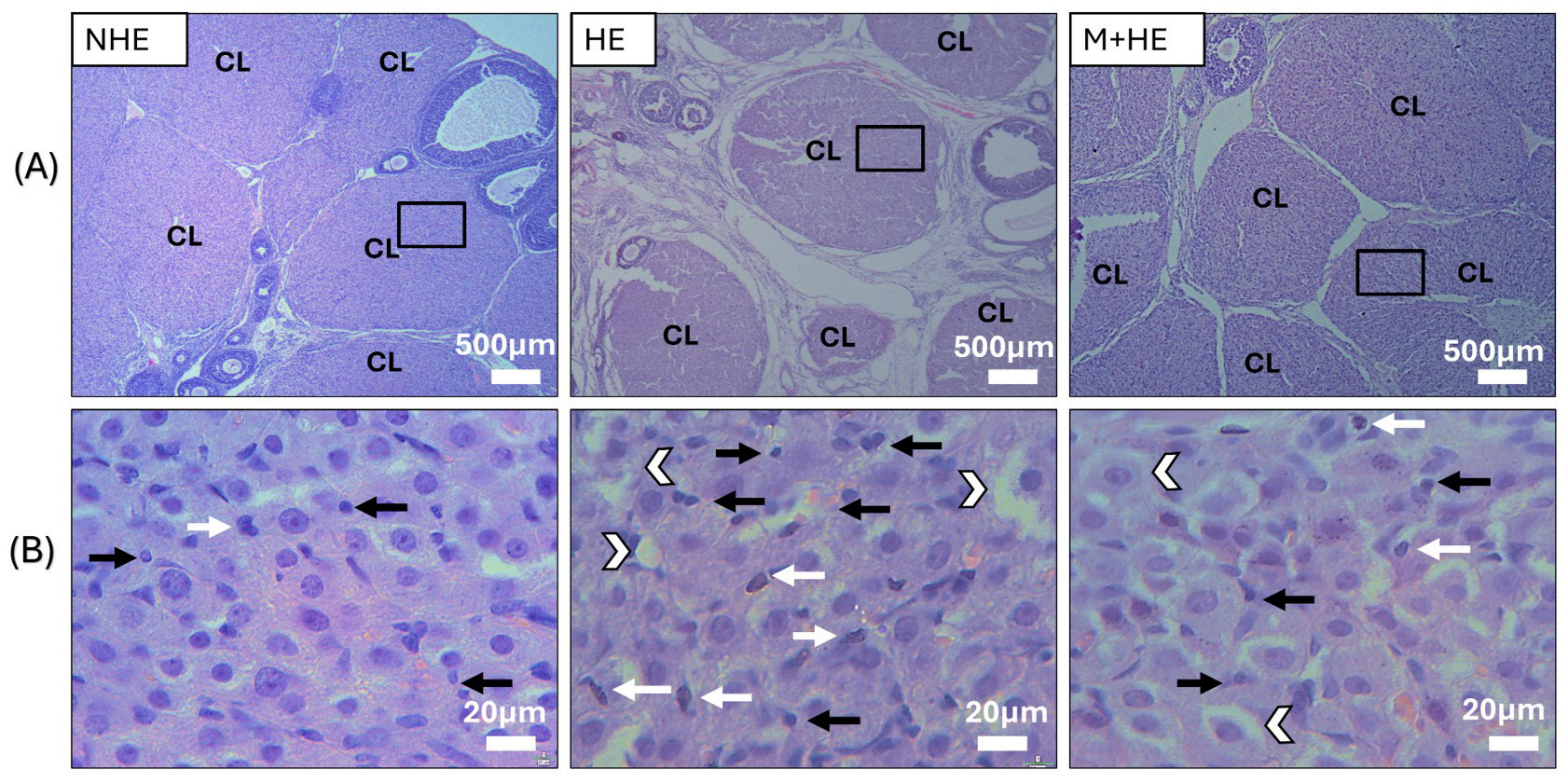
Protective effects of melatonin on the morphological integrity of the CL 24 h post-PGF injection after 7 days of heat exposure. H&E staining was employed to visualize CL changes. (A) The general alterations and degenerative changes in the CL. (B) Specific luteal cell alterations, including vacuolation (white arrowheads), micronuclei formation (black arrows), and apoptotic cells (white arrows). Scale bars represent 500 µm (A) and 20 µm (B).

HE staining revealed notable alterations in the CL of ovarian tissue following 7-day heat exposure. At 24 h post-PGF injection, the HE group exhibited notable structural changes compared with the NHE group. These included vacuolation, characterized by fluid-filled spaces that increased intercellular distances, and hypertrophy of luteal cells (Figure 2A). Additionally, micronuclei formation in luteal cells, a marker of DNA damage, was observed, along with numerous vacuolated cells in the cytoplasm. Apoptotic cells displayed pronounced eosinophilia in the cytoplasm, with shrunken nuclei that appeared dense, crescent-shaped, or fragmented into apoptotic bodies (Figure 2B).

**Figure 3:**
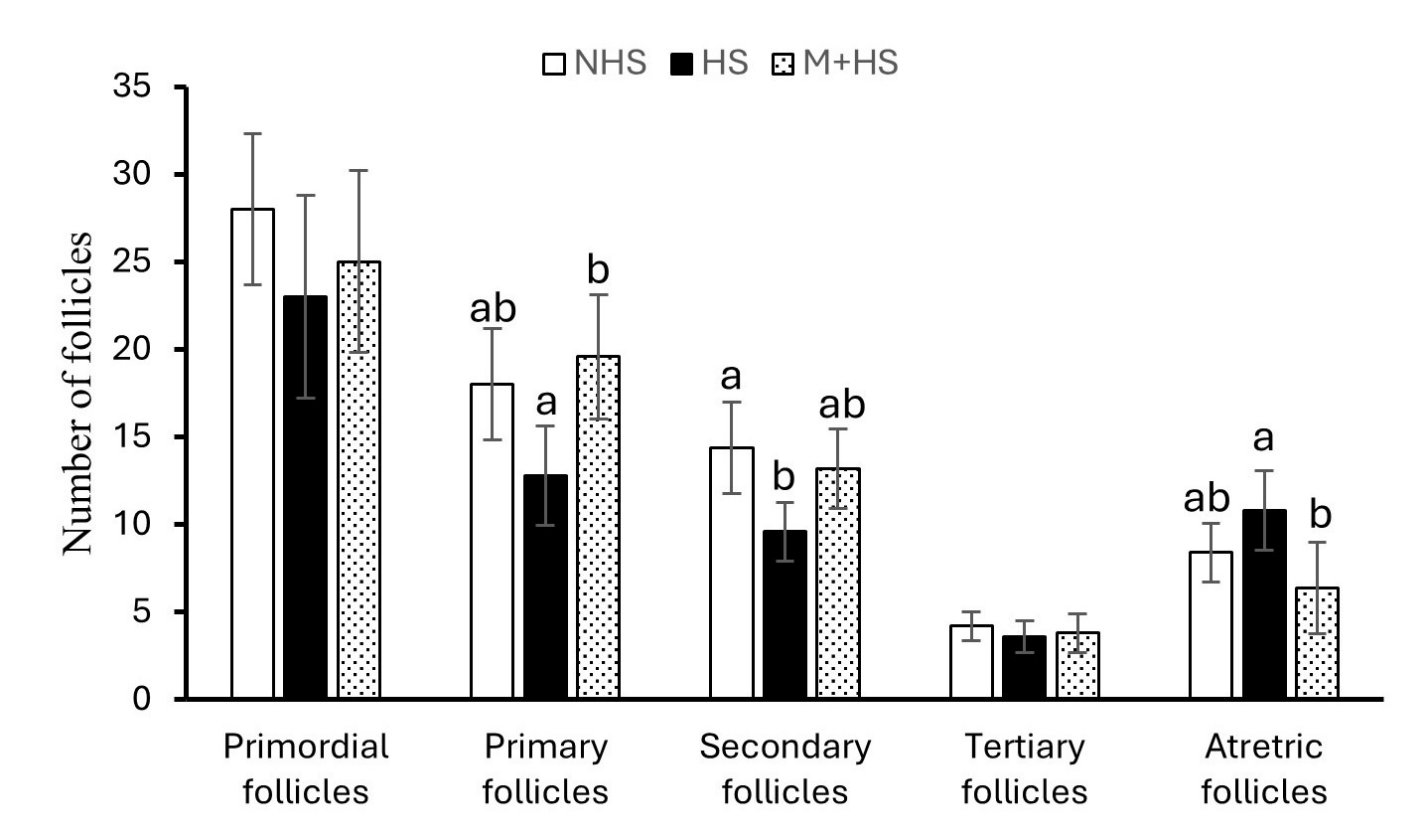
Follicle counts in ovarian tissue under heat exposure and melatonin treatment. The experiment involved the stimulation of ovaries with PMSG and hCG, followed by normal conditions, heat exposure, or melatonin administration to heat-exposed rats. The figure highlights the number of several types of follicles. The data are presented as the mean ± SEM of 3 rats. Different letters denote significant differences between groups (*p* < 0.05).

Histological analysis revealed the distinct effects of heat exposure and melatonin administration on various ovarian follicle populations. In HE group, exposure to heat caused a significant reduction in primary and secondary follicles and a corresponding increase in atretic follicles. In contrast, melatonin treatment significantly mitigated these heat-induced effects, as the M+HE group exhibited a notable increase in the number of primary and secondary follicles (*p* < 0.05, Figure 3). In addition, melatonin treatment led to a significant reduction in atretic follicles compared with the HE group (p < 0.05, Figure 3). The NHE group demonstrated the highest overall follicle count, providing a baseline for normal follicular development in the absence of stress.

### Modulation of melatonin on apoptotic protein expression during PGF-induced luteal regression in heat-exposed rats

**Figure 4:**
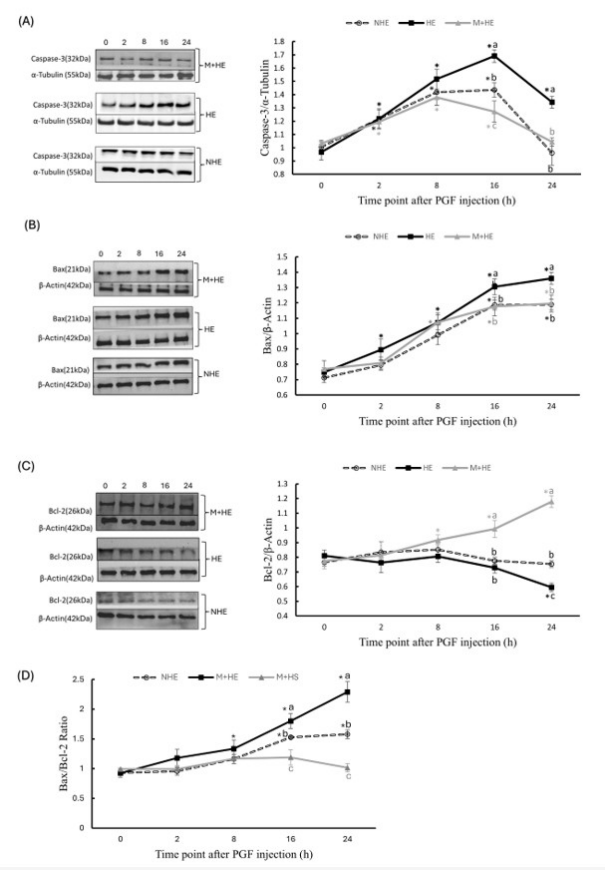
Effects of melatonin on ovarian apoptotic protein expressions after PGF injection in heat-exposed rats. Ovarian tissues were analyzed by Western blot (WB) for the expression of Caspase-3 (A), Bax (B), and Bcl-2 (C). The left panel shows representative WB bands, and the right panel presents quantitative analysis using ImageJ. Panel (D) displays the Bax/Bcl-2 ratio. Results are presented as mean ± SEM from three rats per time point. Within the same group, * indicates a significant difference (*p* < 0.05) compared with 0 h. At the same time point, different letters denote significant differences between groups (*p* < 0.05).

WB analysis demonstrated a time-dependent effect of PGF administration on apoptotic protein expression. Caspase-3 levels gradually increased in all groups until reaching a peak between 8–16 h, followed by a decline at 24 h (Figure 4A). Similarly, Bax expression significantly increased, reaching its highest level at 24 h (Figure 4B). In contrast, Bcl-2 expression remained unchanged in the NHE group post-PGF treatment. However, in the HE group, Bcl-2 levels significantly decreased at 24 h compared with 0 h (*p* < 0.05), whereas in the M+HE group, Bcl-2 expression significantly increased at 8–24 h compared with 0 h (*p* < 0.05, Figure 4C).

Heat stress amplified the PGF-induced apoptotic response, leading to increased Caspase-3 and Bax expression at 16 and 24 h (*p* < 0.05, Figure 4A, 4B), while significantly suppressing Bcl-2 expression at 24 h (*p* < 0.05, Figure 4C) compared with the NHE group. In contrast, melatonin treatment significantly modulated apoptotic protein expression, reducing Caspase-3 levels at 16 and 24 h (*p* < 0.05, Figure 4A) and lowering Bax levels at 16 h (*p* < 0.05) and 24 h (*p* < 0.05, Figure 4B). Furthermore, melatonin significantly increased Bcl-2 expression at 16 and 24 h (*p* < 0.05, Figure 4C) compared with the HE group.

An analysis of Bax/Bcl-2ratios, a key indicator of apoptotic balance, revealed a time-dependent increase in both the NHE and HE groups. Notably, the Bax/Bcl-2 ratio increased after 16 h (*p* < 0.05) and remained elevated until 24 h (*p* < 0.05, Figure 4D) in the HE group compared with the NHE group. In contrast, the Bax/Bcl-2 ratio in the M+HE group decreased significantly at 16 h (*p* < 0.05) and 24 h (*p* < 0.05) compared with the HE group (Figure 4D).

### Effect of melatonin and heat stress on the subcellular structure in luteal steroidogenic cells

**Figure 5:**
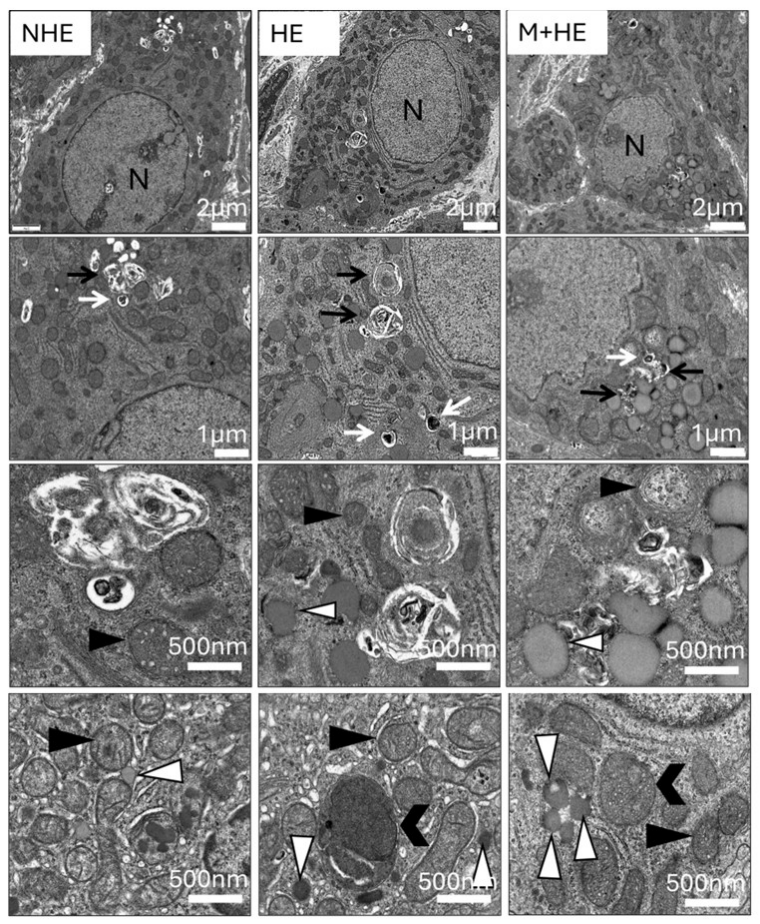
Ultrastructural changes associated with apoptosis in luteal cells at 24 h after PGF injection. TEM reveals various features in luteal cells, including a central nucleus (N), apoptotic bodies (white arrows), autophagosomes (black arrows), normal mitochondria (black triangles), swollen mitochondria (black head arrows), and lipid droplets (white triangles). Scale bars represent 2 μm, 1 μm, and 500 nm.

Transmission electron microscopy was utilized to assess the ultrastructural changes in luteal steroidogenic cells at 24 h post-PGF injection. TEM revealed distinct cellular alterations in response to heat exposure, with melatonin providing partial protection. In the NHE group, cells exhibited well-preserved structures, with intact nuclei and organized mitochondria. The cytoplasmic structure was well preserved, and the mitochondria exhibited densely packed and organized cristae. In the HE group, heat stress caused significant cellular damage, including apoptotic vesicles, condensed chromatin, and mitochondrial alterations such as vacuolization, disorganized cristae, and loss of structural integrity. In the M+HE group, melatonin treatment resulted in fewer mitochondrial alterations, characterized by mild vacuolization and disordered cristae. Additionally, there was an increase in lipid droplets observed in this group (Figure 5).

### Melatonin mitigates the effect of heat stress on luteal apoptosis

**Figure 6:**
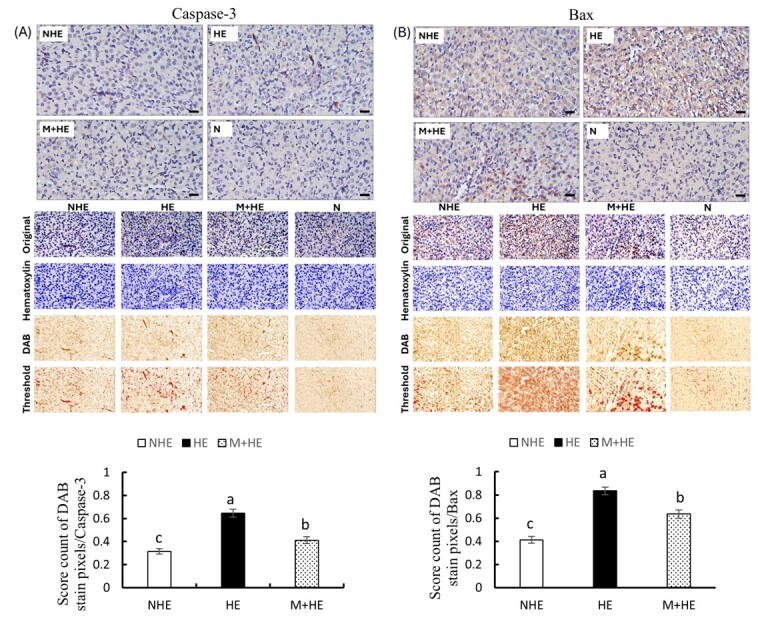
Immunohistochemical staining of apoptotic proteins in luteal cells 24 h post-PGF injection. The upper panel shows representative images for Caspase-3 (A) and Bax (B) staining, with N representing the negative control. The lower panel displays the quantitative analysis of DAB staining scores using ImageJ. Data are presented as means ± SEM from three rats per time point. Different letters denote significant differences (*p* < 0.05). The scale bar represents 20 μm.

To further investigate the melatonin protective role, immunohistochemical analysis was performed to assess luteal cell apoptosis at 24 h post-PGF injection. Results showed that both Bax and Caspase-3 proteins were predominantly expressed in luteal cells across all groups (Figure 6A and 6B). Active Caspase-3 was mainly localized in the nucleus, while Bax was primarily found in the cytoplasm. In the NHE group, the expression of both active Caspase-3 and Bax was weak. However, in the HE group, these proteins were markedly elevated. In contrast, the M+HE group showed significantly reduced immunoreactivity for both proteins compared with the HE group (Figure 6).

## Discussion

In the absence of pregnancy, the timely regression of the CL is critical to enable the initiation of a new reproductive cycle. CL regression, primarily induced by prostaglandin F_2α_ (PGF), involves intricate functional and structural alterations that ultimately leading to apoptosis. This study presents novel insights into the protective role of melatonin against heat stress-induced disruptions during PGF-mediated luteal regression. By assessing physiological responses, structural integrity and apoptotic signaling pathways, our findings demonstrate that melatonin mitigates the effects of heat stress, thereby preserving luteal function. Our results suggest that melatonin impact on PGF-induced luteolysis may be context-dependent. Under normal physiological conditions, melatonin appears to enhance luteolytic signaling, potentially aiding the precise timing of CL regression for subsequent reproductive cycles. However, under heat stress, where luteal cell integrity and function are compromised, melatonin shifts towards a protective role, attenuating excessive apoptosis and preserving ovarian functionality. This dual regulatory capacity suggests that melatonin functions as a responsive modulator of reproductive status, adjusting its role based on environmental stressors. Further exploration of the context-specific actions of melatonin could reveal valuable strategies for mitigating reproductive impairments associated with environmental stress.

Heat stress disrupts homeostasis, notably affecting reproductive tissues (Bei et al., 2020; Soravia et al., 2021). While the primary aim of this study was to investigate the effects of melatonin on luteal regression, we also observed significant benefits in general health parameters, such as body weight. Melatonin appears to help maintain overall homeostasis during heat stress, which likely contributes to the preservation of reproductive function. This is supported by the observation of improved body weight in the melatonin-treated rats, despite the significant weight reductions in the heat-exposed group. While body weight is often considered an indirect marker of overall health, our findings suggest that it may also reflect better reproductive health under heat stress conditions, as evidenced by the improvement in ovarian weight and affected ovarian index in the melatonin-treated rats.

Elevated oxidative stress and apoptosis markers, such as Bax and Caspase-3, further highlight the detrimental effects of heat stress on reproductive performance and ovarian function (Boni, 2019; Collier et al., 2017; Ullah et al., 2019). In our study, melatonin treatment effectively counteracted these disruptions, reinforcing its thermoregulatory and protective properties (Cipolla-Neto and Amaral, 2018; Xu et al., 2022). Specifically, melatonin-treated rats exhibited improved physiological stability, including restored body weight, normalized ovarian metrics, and reduced apoptotic markers. Beyond its effects on luteal regression, melatonin also preserved follicular development, preserving follicular development and reducing atretic follicles under heat stress conditions. Consistent with previous studies, heat stress prolonged luteal regression, induced structural damage to the CL, and inhibited ovulation (Boni, 2019; Collier et al., 2017; Ullah et al., 2019). Our findings suggest that melatonin aids in the regulation of luteal regression by mitigating heat stress- and PGF-induced apoptosis, thereby preserving luteal integrity. This may facilitate a more time-dependent and controlled luteal regression, ultimately supporting reproductive function under stress conditions. The protective effects of melatonin may be linked to neuroendocrine actions (Cruz et al., 2014; Haduch et al., 2016; Jagota and Kalyani, 2010), anxiolytic properties (Esch and Stefano, 2010), and the restoration of circadian rhythms disrupted by heat stress (Abbott et al., 2020; Onaolapo et al., 2017). Further exploration of these mechanistic pathways could provide valuable insights into melatonin role in adaptive reproductive responses to environmental stressors.

Our observations of increased Bax and Caspase-3 expression, as assessed by WB and IHC following PGF injection are consistent with previous reports, demonstrating that PGF induces luteal cell apoptosis by upregulating Bax and activating Caspase-3 (Boice and Bouchier-Hayes, 2020; Jara-Gutiérrez and Baladrón, 2021). The expression patterns of Bcl-2 and Bax in the CL align with prior research, indicating that PGF-induced luteolysis is associated with a significant rise in Bax, whereasBcl-2 remains largely unchanged (Guo et al., 2015; Shah et al., 2014). Heat stress further exacerbates these apoptotic pathways, elevating the demand for energy and oxygen, leading to mitochondrial dysfunction and oxidative stress (Christen et al., 2018). The increased expression of apoptotic markers like Caspase-3 and Bax under heat stress, as demonstrated in our study, is consistent with previous findings in granulosa cells exposed to elevated temperatures (Li et al., 2016). Notably, the ability of melatonin to reduce the expression of Bax and Caspase-3 aligns with its established protective effects against environmental stressors (Al-Shahat et al., 2022; Wang et al., 2021). These findings underscore the role of melatonin in modulating apoptotic pathways during luteal regression, further supporting its potential as a therapeutic agent for mitigating heat-induced reproductive dysfunction.

Apoptosis is a defining feature of luteolysis, characterized by apoptotic bodies, as previously reported in luteal cells across various species (Hernandez et al., 2009). Our histopathological analysis corroborated these findings, with heat-stressed rats showing significant apoptotic features in luteal cells, including DNA fragmentation and nuclear condensation. Moreover, consistent with earlier ultrastructural studies (Jiang et al., 2016), heat stress induced micronuclei formation, a hallmark of chromosomal instability, in the CL. In contrast, melatonin substantially decreased the occurrence of micronuclei, as observed in our study, supporting its protective role against DNA damage. This aligns with earlier findings where melatonin reduced radiation-induced chromosomal damage (Rostami et al., 2016), highlighting its broader genomic protection.

Mitochondria, central to cellular energy production, play a pivotal role in regulating apoptosis. Heat stress compromises mitochondrial integrity through excessive reactive oxygen species (ROS) production, leading to protein denaturation and cellular damage (Abate et al., 2020; Belhadj Slimen et al., 2014). Our electron microscopy visually revealed that heat-stressed rats exhibited damaged mitochondrial membranes and swollen mitochondria, indicative of oxidative stress-induced injury. In contrast, melatonin-treated rats exhibited preserved mitochondrial structure, suggesting a potential protective effect of melatonin on mitochondrial integrity and function and supporting steroidogenesis. This observation aligns with prior findings on the protective effects of melatonin in other cell types subjected to oxidative stress, such as granulosa cells and spermatozoa (Zhao et al., 2021; Zheng et al., 2021), reinforcing its role as a mitochondrial-targeted antioxidant in luteal cells under stress. Further studies, including quantitative analysis, are necessary to validate these observations and draw more definitive conclusions about the effects of melatonin on mitochondrial integrity in heat-stressed luteal cells.

Lipid droplets (LDs) in luteal cells are crucial for cholesterol storage and steroid hormone synthesis, processes that are highly sensitive to disruptions caused by heat stress. In our study, heat-stressed rats exhibited elevated LD accumulation, indicating a possible imbalance in lipid metabolism and impaired steroidogenesis (Emami et al., 2020; Khan et al., 2020b). Interestingly, melatonin-treated rats displayed an even greater accumulation of LDs, suggesting a possible imbalance in lipid metabolism and impaired steroidogenesis capacity under stress. The observed increase in LDs in the melatonin group aligns with roles of melatonin in preserving mitochondrial integrity and supporting steroidogenesis (He et al., 2018). The heightened presence of LDs in melatonin-treated rats highlights the potential of melatonin to protect luteal cells from heat-induced damage and support their steroidogenic function, helping to maintain luteal resilience and function despite environmental challenges. However, further studies with quantitative lipidomic profiling are required to clarify the exact metabolic effects of melatonin in heat-stressed luteal cells.

## Conclusions

This study provides compelling evidence that melatonin alleviates the adverse physiological and apoptotic effects of heat stress on the CL in rats. Heat stress induces physiological disturbances, including reductions in body weight, altered in ovarian morphology, and exacerbating apoptotic processes, as evidenced by the upregulating of pro-apoptotic markers including Bax and Caspase-3. Notably, melatonin administration counteracts these detrimental effects, reducing the expression of apoptotic proteins, and preserving cellular integrity. These findings highlight melatonin potential as a therapeutic agent for mitigating heat stress-induced reproductive dysfunction, offering a promising avenue for preserving reproductive health under environmental stress conditions.

## Data Availability

Research data is only available upon request.
